# Life satisfaction after traumatic brain injury: comparison of ratings with the Life Satisfaction Questionnaire (LiSat-11) and the Satisfaction With Life Scale (SWLS)

**DOI:** 10.1186/s12955-016-0405-y

**Published:** 2016-01-15

**Authors:** Lars Jacobsson, Jan Lexell

**Affiliations:** Department of Health Sciences, Lund University, Lund, Sweden; Department of Rehabilitation Medicine, Sunderby Hospital, Luleå, Sweden; Department of Health Sciences, Luleå University of Technology, Luleå, Sweden; Department of Rehabilitation Medicine, Skåne University Hospital in Lund, Lund, Sweden

**Keywords:** Brain injuries, Outcome assessment, Quality of life, Questionnaires, Rehabilitation

## Abstract

**Background:**

An optimal life satisfaction (LS) is considered an important long-term outcome after a traumatic brain injury (TBI). It is, however, not clear to what extent a single instrument captures all aspects of LS, and different instruments may be needed to comprehensively describe LS. The aim of this study was to compare self-ratings of life satisfaction after a TBI with two commonly used instruments.

**Methods:**

Life Satisfaction Questionnaire (LiSat-11), comprising eleven items and Satisfaction With Life Scale (SWLS), comprising five items, were administered to 67 individuals (51 men and 16 women). Secondary analysis of data collected as part of a survey of individuals with TBI 6 to 15 years post TBI.

**Results:**

Item 1 in LiSat-11 (‘Life as a whole’) and the total SWLS score was strongly correlated (Spearman’s rho = 0.66; *p <* 0.001). The total score in SWLS had the strongest correlation with items in LiSat-11. All items in LiSat-11, except ‘Family life’ and ‘Partner relationship’, were moderately to strongly correlated with items in SWLS. The item ‘Partner relationship’ in LiSat-11 did not correlate with any of the items in SWLS or the total score. The item ‘If I could live my life over, I would change nothing’ in SWLS had the weakest correlations with items in LiSat-11. Items ‘Vocation’ and ‘Leisure’ in LISat-11 were most strongly correlated with items in SWLS, whereas the item ‘ADL’ in LiSat-11 was more weakly correlated with items in SWLS.

**Conclusions:**

The strength of the relationships implies that the two instruments assess similar but not identical aspects of LS and therefore complement each other when it is rated.

## Introduction

A high level of life satisfaction (LS) is considered an important outcome of rehabilitation and a long-term endpoint after a traumatic brain injury (TBI). LS is defined as an individual’s contentment with life. It is commonly referred to as the degree of an individual’s subjective appraisal if his or her aspirations or goals and achievements have been accomplished [1, 2].

Two internationally validated instruments assessing LS after TBI are the Life Satisfaction Questionnaire (LiSat-11) [3] and the Satisfaction With Life Scale (SWLS) [4] . Even though these two instruments assess LS, there are notable differences between them. LiSat-11 assesses global LS in 1 item and domain-specific LS in10 items that are expected to be vital aspects of one’s LS [5]. SWLS comprises items that asses an individual’s subjective evaluations of ideal life, a wish for change, and satisfaction with the past and current situation, and is summated as a total score providing a global measure of an individual’s LS [4]. Regardless of which instrument that is used, it has been consistently shown that LS is generally lower in individuals shortly after as well as many years after a TBI [6]. However, it is not clear to what extent a single instrument captures all aspects of LS, and different instruments may be needed to comprehensively describe LS. To the best of our knowledge, no study has pursued a face-to-face comparison of ratings with instruments assessing LS in people with TBI.

The aim of this study was to compare ratings of LS using the two instruments LiSat-11 and SWLS in individuals with TBI. This is a secondary analysis of data on LS, using LiSat-11 [5] and SWLS [4], collected as part of the larger survey of individuals with TBI in northern Sweden 6 to 15 years post TBI [7].

## Methods

### Participants

Participants were obtained from a sample of 332 individuals with a computed tomography (CT) verified TBI and brain injury symptoms who had been transferred to the only Neurosurgical Clinic in the region for neurosurgical care during the period 1 January 1992 to 31 December 2001 [8]. From the population of 332 individuals, 106 met the inclusion criteria of being between 18 and 65 years of age at the time of data collection in year 2007, and a total of 88 (83 % of the 106 potential participants) were assessed on average 10 years post injury regarding their functioning and disability [9]. Of these, sixty-seven individuals (51 men and 16 women) had recovered to such a degree that they could rate their LS using the two instruments [6] and data from these ratings were used in the present study. All data were collected by the first author during individual consultations. Sixty individuals completed the questionnaires by themselves, three individuals had a close relative present but completed the questionnaires independently and four individuals had assistance reading and understanding some of the items in the questionnaires but then completed them by themselves. The mean age of the 67 participants was 44 years (SD = 13; range 19-64 years). Thirty-two had sustained a mild TBI and 35 a moderate-to severe TBI. No significant differences were found between the 67 participants and the 39 (out of 106) and the 21 (out of 88) non-participants, respectively, regarding sex, age at time for injury, injury severity or time post-injury. The study was approved by the Regional Ethical Review Board, Umeå, Sweden (Dnr 06-013 M).

### Questionnaires

The LiSat-11 (11-item Life Satisfaction Questionnaire) [3, 5] assesses global satisfaction with life in one item and domain-specific satisfaction in ten items on six response levels, from ‘very satisfied’ (response option 6) to ‘very dissatisfied’ (response option 1). LiSat-11, an extension of LiSat-9 [10], has been found to be valid for the population at large [3, 5]. The 11 items can also be dichotomized as ‘satisfied’ (very satisfied and satisfied, response option 5 and 6) and ‘not satisfied’ (from rather satisfied to very dissatisfied, response option 1-4) [5]. In the present study, data were not dichotomized and all data were used in the analysis.

The SWLS (Satisfaction With Life Scale) provides a global measure of satisfaction with life as an overall summation of a person’s LS [4]. It consists of five questions rated on a 7-point Likert scale, from ‘strongly agree’ (response option 7) to ‘strongly disagree’ (response option 1). In agreement with previous studies the scores are summed to a total score ranging from 5 to 35. A score of 20 represents the midpoint between satisfied and dissatisfied with life.

### Statistical analysis

All analyses were performed using IBM SPSS statistics 22.0 software (IBM Corporation, Armonk, New York. As both scales can be considered ordinal scales, non-parametric statistics were used and correlations between items in SWLS and LiSat-11 were analysed with the Spearman’s rank correlation (rho).

## Results

In Table 1, data on LiSat-11 for the 67 participants are presented. Many of the participants were to some degree satisfied with life as a whole and with all 10 domains of LS. In Table 2, data on SWLS for the 67 participants are presented. The mean for each item varied between 4.03 (Item 1) to 4.72 (Item 3). The mean total score was 21.3 (SD 7.9), indicating that the participants had an average LS.

**Table 1:** Percentages of self-reported life satisfaction in 67 Swedish individuals with a traumatic brain injury

	11-item Life Satisfaction Questionnaire (LiSat-11)					
	Very dissatisfied (1)	Dissatisfied (2)	Rather dissatisfied (3)	Rather satisfied (4)	Satisfied (5)	Very satisfied (6)
1. Life as a whole	5	6	6	31	34	18
2. Vocation	25	5	10	18	21	21
3. Economy	10	9	10	34	19	16
4. Leisure	8	6	16	27	24	19
5. Contacts	5	9	2	27	30	28
6. Sexual life	18	6	8	22	33	13
7. ADL	5	3	5	6	22	60
8. Family life^a^ (n=41)	0	2	2	7	17	71
9. Partner relationship^b^ (n=35)	0	0	6	6	29	60
10. Somatic health	13	9	10	25	33	9
11. Psychological health	5	5	8	15	37	31

^a^Those that reported to have a family

^b^Those with partner

**Table 2:** Percentages of self-reported life satisfaction in 67 Swedish individuals with a traumatic brain injury

	Satisfaction With Life Scale (SWLS)						
	Strongly disagree (1)	Disagree (2)	Slightly disagree (3)	Neither agree nor disagree (4)	Slightly agree (5)	Agree (6)	Strongly agree (7)
1. In most ways my life is close to my ideal	16	13	8	13	18	25	6
2. The conditions of my life are excellent	12	12	16	8	30	10	12
3. I am satisfied with my life	10	5	9	12	19	33	12
4. So far I have gotten the important things I want in life	19	9	10	5	19	21	16
5. If I could live my life over, I would change almost nothing	18	12	9	9	13	22	16

There were significant correlations (*p <* 0.001 to *p <* 0.05); Spearman’s rho = 0.27 to 0.67) between 34 of the 55 items in LiSat-11. The item ‘Partner relationship’ was not significantly correlated with any of the other items in LiSat-11, and the item ‘Family life’ was significantly correlated with only 3 of the 11 items. All 5 items in SWLS were significantly correlated *p <* 0.01 to *p <* 0.001; Spearman’s rho = 0.35 to 0.81).

In Table 3, the results of the correlations between items in the two instruments are presented. The global measure in LiSat-11 (‘Life as a whole’) and the total SWLS score was strongly correlated (Spearman’s rho = 0.66; *p <* 0.001). Overall, the total score in SWLS had the strongest correlation with items in LiSat-11. Generally, all items in LiSat-11, except ‘Family life’ and ‘Partner relationship’, were moderately to strongly correlated with items in SWLS. The item ‘Partner relationship’ in LiSat-11 did not correlate with any of the items in SWLS or the total score. Item ‘If I could live my life over, I would change nothing’ in SWLS had the weakest correlations with items in LiSat-11. Items ‘Vocation’ and ‘Leisure’ were most strongly correlated with the items in SWLS, whereas the item ‘ADL’ in LiSat-11 was more weakly correlated with items in SWLS.

**Table 3:** Relationship between items of LiSat-11 and items in SWLS in 67 individuals with traumatic brain injury

	LiSat-11 (11-item Life Satisfaction Questionnaire)										
SWLS (Satisfaction With Life Scale)	1. Life as a whole	2. Vocation	3. Economy	4. Leisure	5. Contacts	6. Sexual life	7. ADL	8. Family life^a^ (n=41)	9. Partner relationship^b^ (n=35)	10. Somatic health	11. Psychol health
1. In most ways my life is close to my ideal	0.59^e^	0.58^e^	0.35^d^	0.61^e^	0.32^d^	0.50^e^	0.36^d^	0.30	-0.15	0.44^e^	0.35^d^
2. The conditions of my life are excellent	0.65^e^	0.66^e^	0.44^e^	0.63^e^	0.43^e^	0.47^e^	0.46^e^	0.42^c^	-0.18	0.58^e^	0.41^d^
3. I am satisfied with my life	0.57^e^	0.57^e^	0.39^d^	0.56^e^	0.53^e^	0.26^c^	0.39^d^	0.45^d^	0.10	0.44^d^	0.45^e^
4. So far I have gotten the important things I want in life	0.47^e^	0.47^e^	0.46^e^	0.53^e^	0.42^e^	0.39^d^	0.33^d^	0.41^d^	0.25	0.50^e^	0.21
5. If I could live my life over, I would change almost nothing	0.39^d^	0.29^c^	0.41^d^	0.32^d^	0.41^d^	0.36^d^	0.18	0.08	0.22	0.28^c^	0.34^d^
Total score	0.66^e^	0.64^e^	0.51^e^	0.65^e^	0.51^e^	0.51^e^	0.43^e^	0.44^d^	0.13	0.57^e^	0.44^e^

^a^ & ^b^ Item rated by those with a) family, and b) partner

Correlation (Spearman’s rank correlation) is significant at the 5% (^c^), 1% (^d^) and 0.1% (^e^) levels

## Discussion

Life satisfaction is often emphasized in modern rehabilitation and in outcome research of lifelong disabilities, such as TBI. Different instruments are available to assess LS, but as LS is multifaceted, it can be assumed that one instrument alone does not capture all aspects of LS. Consequently, there is a need for an in-depth knowledge of how different LS instruments are related. This study is, to the best of our knowledge, the first study that has pursued a face-to-face comparison of two commonly used instruments assessing LS in people with TBI. Overall, we found a moderate correlation between most items in the two instruments, which indicates that they measure similar but not the same aspects of LS.

As expected, Item 1 in LiSat-11 (‘Life as a whole’) and the total SWLS score were strongly related (Spearman’s r = 0.66; *p <* 0.001). They can both be considered to represent some form of global measure of satisfaction with life. One previous study has found a good convergent validity with other LS instruments in people with spinal cord injury [11]. Moreover, the total score in SWLS had the strongest correlation with the items in LiSat-11, indicating that it can be regarded as a good representation of satisfaction with life and thereby useful studying LS in people with lifelong disabilities after TBI. This is in agreement with Corrigan et al. [12] who stated that the SWLS total score is useful for group comparisons in research or program evaluation.

The item ‘Family life’ in LiSat-11 was only weakly correlated with items in SWLS, and the item ‘Partner relationship’ did not correlate with any of the items in SWLS or the total score. Both these items had the highest degree of satisfaction – 88 % and 89 %, rated these items as satisfied or very satisfied, respectively – which most likely explains the low correlation between items in SWLS. Thus, it seems that having a family life and/or a partner is very important for LS. However, only those who report that they have a family rate this item, which should be considered when these items are interpreted.

Item 5 in SWLS (‘If I could live my life over, I would change almost nothing’) generally had the lowest correlation with items in LiSat-11. This was noted in the review of SWLS [12], where the authors stated that item 1 is consistently most associated with the total score and latent factor, while item 5 is least frequently associated. The authors also stated that it would be essential to understand what life domains are important to the individual and the basis of the evaluation made. Thus, some caution should always be applied when the results of single items in LS instruments are interpreted and generalised.

The items ‘Vocation’ and ‘Leisure’ in LiSat-11 were generally strongly correlated with items 1 to 4 in SWLS. In fact, satisfaction with ‘Vocation’ was the item most strongly correlated with a specific item in SWLS (Item 2). This supports previous studies that reported that people with TBI who are in gainful employment, regardless of the severity of their injury, are more satisfied with life than those who have a disability pension [13]. Similarly, participating in meaningful leisure activities have also been noted to lead to a high degree of LS [13].

The results of this study have some general implications for clinical research and give rise to some aspects that need to be more carefully addressed. One is the content of the items in relation to the overarching research question. In situations where the purpose is to evaluate changes over time or effects of rehabilitation interventions, the global measure of SWLS can be considered together with item 1 in LiSat-11. On the other hand, if the purpose is to explore domains that are vital aspects of one’s LS and compare those, the LiSat-11 may be a better instrument.

One limitation to this study is the relatively small and selected sample. Therefore, the results cannot be generalised to all people with TBI. It is known that cognitive impairments can affect the ability to respond to LS questionnaires. Thus, some of those with a severe TBI may have had difficulties to rate their life situation. Although none of the participants raised any concerns during the ratings, the inferences of this study should be treated with some caution.

In conclusion, we found significant correlations between most items in the two LS instruments. The strength of the relationships implies that they assess similar but not identical aspects. The two instruments can be used simultaneously and complement each other and thereby cover life satisfaction in a broader sense.
